# Untapped Potential: A Qualitative Study of a Hospital-Based Dengue Surveillance System

**DOI:** 10.4269/ajtmh.19-0719

**Published:** 2020-05-11

**Authors:** Sulistyawati Sulistyawati, Maria Nilsson, Marlita Putri Ekasari, Surahma Asti Mulasari, Tri Wahyuni Sukesi, Retna Siwi Padmawati, Åsa Holmner

**Affiliations:** 1 Department of Public Health, Universitas Ahmad Dahlan, Yogyakarta, Indonesia;; 2 Department of Epidemiology and Global Health, Umeå University, Umeå, Sweden;; 3 Laboratory of Pharmacy Management and Community Pharmacy, Department of Pharmaceutics, Universitas Gadjah Mada, Yogyakarta, Indonesia;; 4 Department of Health Behaviour, Environmental, and Social Medicine, Universitas Gadjah Mada, Yogyakarta, Indonesia;; 5 Center of Health Behavior and Promotion, Faculty of Medicine, Public Health and Nursing, Universitas Gadjah Mada, Yogyakarta, Indonesia;; 6 Department of Radiation Sciences, Umeå University, Umeå, Sweden

## Abstract

The incidence and geographical distribution of dengue fever has increased in recent decades. The actual disease burden is unknown owing to frequent underreporting and misclassification of cases. A well-functioning system for diagnosing, treating, and reporting cases is of prime importance as disease statistics is the foundation for decisions aiming to control the disease. This study aimed to explore the hospital-based disease surveillance system in Yogyakarta, a dengue-endemic region on Java, Indonesia. Semi-structured interviews were performed with 16 informants from four hospitals, including five general practitioners, three internists, four pediatricians, and four administrative staff working with administration relating to dengue diagnostics and reporting. Data were analyzed using content analysis. A theme arose from the analysis “Dengue surveillance stands and falls by the rigor of the health system.” The theme, and underlying categories and subcategories, describes a surveillance system that in the best-case scenario works well and is likely to produce reliable dengue case data. However, there is a lack of synchronization between regulations and guidelines in different hospitals and some friction between regulatory bodies and the care provider. Knowledge among the staff appears to vary, and many clinical and financial decisions are made rather arbitrarily, which ultimately might lead to unequal health service delivery. In conclusion, the dengue surveillance system under study could improve further, particularly by ensuring that all regulations and recommended procedures are standardized and that all staff are given the best opportunity to stay updated on dengue-related matters, clinical as well as regulatory, on a regular basis.

## INTRODUCTION

The global burden of dengue fever (DF) is unknown, partly because of underreporting and misclassification of dengue cases,^1,2^ with negative consequences for disease prevention and control. Based on official data from WHO member states, the number of dengue cases exceeded 3.34 millions in 2016, but the numbers are predicted to be closer to 390 million cases per year, of which 67–136 million manifest clinically.^3^ The escalation of the spread of dengue is closely associated with population growth, urbanization, and shortcomings in environmental management.^4^ Moreover, there is increasing evidence that climate change is affecting the incidence rate and the spatial distribution of the disease, in addition to changing the timing and duration of disease outbreaks.^5,6^

Various methods have been applied to establish more accurate dengue estimates,^7,8^ and some have identified components critical for an efficient dengue surveillance system.^9^ The mechanisms behind dengue underreporting, however, have attracted limited attention according to the literature. Dengue has proven a challenge to categorize,^10^ and new case classifications were launched by the WHO in 2009 to improve triage and case management. Today, dengue is to be classified into dengue with or without warning signs, and severe dengue, instead of the old classifications DF, dengue hemorrhagic fever (DHF), and dengue shock syndrome (DSS) stipulated by previous WHO frameworks. As dengue produces a broad spectrum of symptoms, recommendations are that diagnosis is confirmed by virus isolation and serotype identification, viral antigen ELISA tests, or non-structural protein 1 (NS1) antigen rapid test, although access to diagnostic tools varies significantly across dengue-endemic regions.^11^ In Indonesia, the usage of NS1 antigen in diagnostics is not an official requirement but is sometimes used on patient requests or in special circumstances, such as in Yogyakarta city where the (previously called the Eliminate Dengue program) provides NS1 rapid test equipment to all primary healthcare centers and selected hospitals.

This article set out to investigate the current disease surveillance system in Yogyakarta city, Indonesia, which has been a dengue-endemic region for years.^12^ The city has a population of more than 400,000 people,^13,14^ and health services are provided by 11 public hospitals, 11 specialist hospitals,^15^ and 18 primary healthcare centers. The responsibility for planning, managing, and allocating funding for public health services primarily lies with the local government at the district level. The district health office (DHO) is thus the main regulator of dengue surveillance, prevention, and control and supervises public as well as private hospitals. In 2014, Indonesia introduced a new national health insurance system, administered by the Social Security Administering Body (BPJS), to offer basic health services for all citizens, regardless of the income level.^16,17^ In parallel, a new referral system was established, which stipulates that, for the insurance to be valid, primary care shall handle all non-emergent cases and only refer to upper-level hospitals when needed.

The enactment of BPJS has revolutionized health care for many people in Indonesia, particularly the poor. Currently, BPJS has three premium levels: basic, medium, and high. The premium level directly regulates the level of care that is covered.^18^ Before the BPJS program, public insurances were only provided to government employees, whereas everyone else had to purchase a private insurance. Those self-employed, or without employment, still need to finance their insurance, which is mandatory for every citizen in the country, but there are also programs available for those without financial capacity. The BPJS funding is primarily allocated to curative care activities and not to preventive care and public health initiatives.^19^

In Yogyakarta, disease statistics is based primarily on hospital data, although suspected and confirmed cases from all care levels shall be reported within 24 hours in line with WHO regulations. A case report containing the individual patient profile and clinical characteristics^20^ is to be prepared for all confirmed cases and delivered using a form called hospital early alertness report (KDRS). The hospital is obliged to release two KDRS documents: one to the local health authority within 24 hours and one to the patient. The first report will be forwarded to the nearest public health center, which is obliged to conduct an epidemiology investigation in the infected persons’ neighborhood.^21^ The second report is to be handed over to the local village administration to increase awareness in the local community.

The aim of this study was to explore strengths, weaknesses, and potential gaps in the hospital-based dengue surveillance system in Yogyakarta from the viewpoint of hospital staff involved in the process of diagnosing, treating, and reporting dengue cases to the local health authorities.

## METHODS

### Study design.

A qualitative research method was used to explore the hospital-based dengue surveillance system as perceived by staff working with dengue diagnostics, treatment, and reporting. A qualitative flexible research design with purposive sampling of informants was regarded the best means to gain in-depth insight into this topic. Four hospitals were selected by the research team to represent the hospitals in Yogyakarta: two public and two private hospitals.

Semi-structured in-depth interviews were performed with staff involved in dengue diagnostics and reporting. Qualitative content analysis was used to further explore the results and identify strengths, weaknesses, and improvement potential in the system under study.

### Study location and sampling.

This study was conducted in four hospitals in Yogyakarta, Indonesia. Permission was obtained from the Yogyakarta Health Office, and participants were purposively selected based on the following criteria: 1) had worked in the hospital for more than 1 year, 2) was a health professional, and 3) was involved in dengue case management and reporting. The informants represented professions with direct experiences of the topics under study, namely pediatricians, general practitioners, internists, or medical record officers—one participant per profession and hospital. In the first step of recruiting informants, the research group sent information to the hospital management, including inclusion criteria and a copy of the research permission. The first author thereafter contacted the informants who were appointed by the hospital management to schedule individual interviews. Hospitals selected were both private and public state hospitals, so as to represent staff working in different types of hospitals under different conditions in Yogyakarta.

### Data collection.

In-depth interviews were conducted in four hospitals in Yogyakarta, Indonesia, between January 2017 and September 2017, using a semi-structured interview guide. The use of in-depth interviews made it possible to explore the topics of interest with the informants in terms of depth, which gave richness in data. A flexible design was used in data collection, allowing an iterative process where new ideas from one informant could be further explored in the following interviews. The interview guide was developed based on the results from a survey performed in 2014 among general practitioners employed in all major hospitals in Yogyakarta, including the four hospitals involved in this study (unpublished results). The survey assessed people’s knowledge, attitudes, and practices with respect to dengue diagnostics and reporting, and implied weaknesses in several areas, including adherence to the 24-hour reporting regulations and knowledge about the disease, which may influence the surveillance quality. Hence, the interview guide included questions regarding dengue symptoms and warnings signs, the process of dengue diagnostics and perceived difficulties and weaknesses, guidelines used, dengue case reporting routines, association of dengue management with the national insurance system, and ideas for the improvement of the management and reporting system. The interviews lasted between 60 and 90 minutes and were conducted in the respondent’s workplace in a room where it was possible for the interviews to be conducted without disturbance from others. One participant could not join because of lack of time, and one hospital sent two general practitioners (GPs) instead of one to the interview. The two GPs were interviewed together at the same time on one occasion, which meant that 15 interviews were performed with a total of 16 informants. Written informed consent was collected from the informants before the interviews, including informant permission to digitally record the interviews. Field notes were taken by the interviewer during the interviews and were afterward summarized and read out loud to the informants, who were asked for confirmation. The first author of this article, with an understanding of the dengue surveillance system in Yogyakarta, conducted all interviews in the Bahasa Indonesian language. Follow-up questions were posed to probe further for information that could increase the understanding of the area under research. Saturation was reached after 15 interviews.

### Analysis.

All the interviews were transcribed verbatim in the original language (Bahasa Indonesia) for subsequent analysis. A qualitative content analysis was performed, as described by Graneheim and Lundman,^22^ to analyze the data, focusing on the manifest content. The analysis started by identifying meaning units that related to the same meaning and corresponded to fulfilling the aim of the study. Then, meaning units were shortened by condensation and followed by a coding process where they were labeled with codes possible to understand in relation to the context. In the next phase, similar codes were grouped and formed to subcategories, which were grouped into categories sharing commonalities of content and meaning. In the final step, we developed a theme that both connected and cut across the different categories, giving joint threads of meaning. The entire coding was performed in Bahasa Indonesia, and from the subcategory level, the data were translated to English. Four researchers were included in the initial coding process (S. S., M. P. E., T. W. S., and S. A. M.). Regular meetings were held during the process to develop the codes and reach agreement.

**Figure 1.**
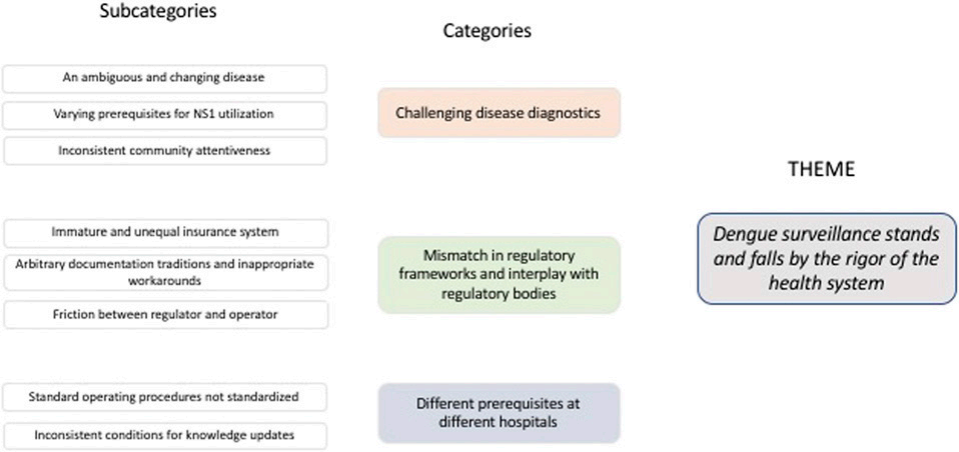
Illustration of the result of the analysis, including overarching theme, categories, and subcategories. This figure appears in color at www.ajtmh.org.

**Figure 2.**
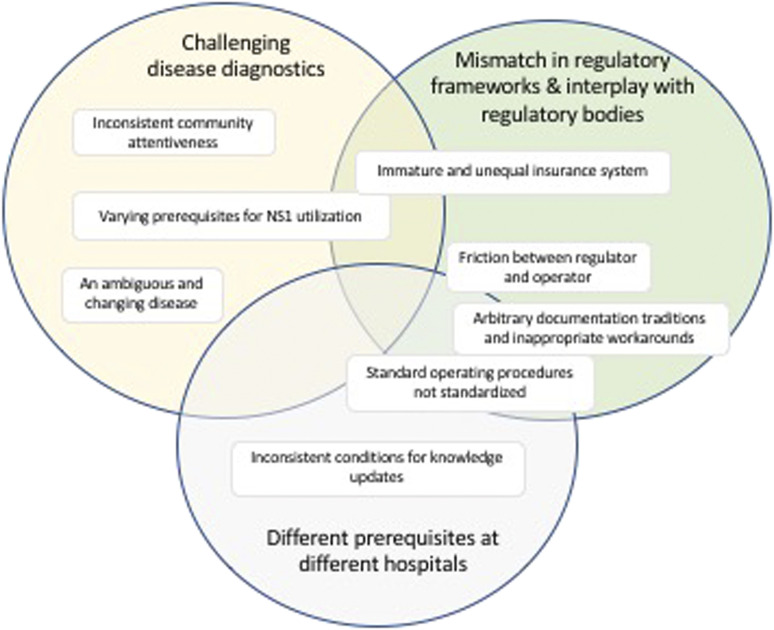
Schematic illustration of the outcome of the analysis as described by three categories and their respective subcategories. Some subcategories are more connected than others, as illustrated by their position in the figure. This figure appears in color at www.ajtmh.org.

Trustworthiness was achieved in the preparation, organization, and reporting, following a checklist provided by Forman and Damschroder in 2007.^23^ In the analysis and reporting of the study, Guba and Lincoln’s criteria for credibility, transferability, dependability, and confirmability were followed. Credibility was achieved through a systematic approach for data collection and analysis. Figures 1 and 2 show an example of how researchers worked with the analytical process from meaning units to theme, giving a possibility to judge the credibility of the findings. We strived for transferability by sampling informants from hospitals with diverse conditions in terms of being private and public. We achieved dependability by involving a third researcher in the analysis who read all the interviews, a researcher who had not been involved in the previous coding process. We achieved confirmability using audio recordings and by the use of direct quotes in the findings section. Triangulation was performed using data source approach by cross-checking the data among the informants.^24^ Credibility was also positively impacted by triangulation in terms of expertise. All researchers work in public health and were actively involved in every research phase. The first author (S. S.) is a researcher in public health with an interest in DF epidemiology, the second author (M. N.) is a researcher in public health with interest in climate change and health research, the third author (M. P. E.) is a researcher with a pharmacist background who is focused on international health, the fourth and fifth authors (S. A. M. and T. W. S.) have a public health background with interest in health policy and dengue, and the sixth author (R. S. P.) comes with a public health background and experience in health system research. Last, the seventh author (A. H.) is a researcher with a background in bioscience, with research in infectious diseases and sustainable health systems. S. S., M. P. E., S. A. M., T. W. S., and R. S. P. are of Indonesian residence and all knowledgeable of Javanese culture.

The Ethical Review Board of Universitas Ahmad Dahlan, Yogyakarta Indonesia, granted ethical approval before the start of the study (no. 011509070). According to custom, the hospitals were recommended by the City Health Office, and the informants were appointed by the hospital management, which potentially impacted the protection of confidentiality and anonymity. However, the researchers took every step possible to protect the ethical principles when presenting the results so that no informant could be identified—for example, by masking content in the text. The informants gave rich descriptions in face-to-face interviews. The informants gave their informed consent to participate and were aware of their right to decline participation or withdraw at any time when being interviewed.

## RESULTS

Fifteen in-depth interviews were conducted with 16 informants recruited from four hospitals in Yogyakarta, two public and two privates. Nine respondents worked in the public sector and seven in the private. Eleven of the informants were females and five were males. The informants had worked in their current workplace for 3–5 years and held positions as GP (5), internist (3), pediatrician (4), or medical record officer (4).

Our analysis resulted in 11 subcategories grouped into three major categories, which together form a theme named “Dengue surveillance stands and falls by the rigor of the health system.” This theme represents a health system with weaknesses in three distinct areas, captured by the three categories: “Challenging disease diagnostics,” addressing the inherent challenges of diagnosing dengue correctly; “Mismatch in regulatory frameworks and interplay with regulatory bodies,” which describes challenges in dengue diagnostics and reporting imposed by regulations, regulatory frameworks, and interaction with regulatory bodies; and “Different prerequisites at different hospitals,” which signifies disparities caused by local management, attitudes, regulations, and variable quality of staff*.* The analysis revealed a lot of good examples and experiences, and the material clearly speaks of a potential that is not fully explored in all hospitals under study. The theme, categories, and subcategories are presented in Figure 1.

There is a certain overlap in the material owing to the complexity of the system under study. The subcategories have been abstracted into distinct categories; however, we have found close interactions between subcategories that belong to different categories. We have referred to these connections in the text, and the touchpoints are illustrated in Figure 2 for clarification and further elaborated on in the discussion.

The following section describes the categories and their subcategories (in bold). Quotes are chosen as examples of the codes underlying the analysis and are presented in italics.

### Challenging disease diagnostics.

This category is composed of the subcategories “**an ambiguous and changing disease**,” “**varying prerequisites for NS1 utilization,**” and “**inconsistent community attentiveness**.” They represent the challenges of diagnosing dengue, mainly owing to the inherent nature of the disease and people’s ignorance or unawareness of the disease’s manifestations.

The first subcategory “**an ambiguous and changing disease**” represents not only the informants’ views that dengue has similar symptoms to many other diseases but also that the pattern of disease has changed in recent years. Most clinicians talked about the early symptoms of the disease development as being very general and easily confused with other infectious diseases:

… because of the disease journey. It is fever. Fever in children has many DDs [differential diagnoses]. Common cold is also fever. Then typhoid is also fever. Even measles, rubella. The pattern is the same. Fever for four days, temperature decrease, appearance of a rash. (Pediatrician 4)

…meanwhile dengue and Leptospira symptoms are similar at the beginning of the infection….this leads to some missed cases… (Internist 3)

The many differential diagnoses (DDs) were also considered a problem when the patient was referred from another hospital, sometimes with an incorrect assessment or diagnosis:

The clinical condition was not dengue and this often happens. So, for me, when it comes to referred (patients), we must do a reassessment. (Internist 3)

Another phenomenon brought up by the informants was the so-called expanded dengue, a concept coined by the WHO to explain atypical findings and deviations from the common disease pattern.^25^ Several clinicians reported that they have met patients with expanded dengue that displayed uncharacteristic disease development and symptoms. This was said to require more attention from the healthcare attendants to prevent misdiagnosis, or even death, as this has been the outcome occasionally, according to one informant. In addition, virus mutations were mentioned as the cause for the atypical presentation seen in recent years:

Several years ago, dengue patients came with symptoms such as fever for three days, headache, nausea, and sickness. But now, we find dengue anomalies. (Pediatrician 4)

Currently, we are dealing with expanded dengue. Perhaps this phenomenon makes it difficult for us to recognize dengue, or we can say that diagnosing dengue without classical symptoms is a challenge. So, in my opinion, if we found a patient with classical dengue, it would be easy to diagnose. (Pediatrician 1)

Yes. Dengue’s DNA mutation is high. I do not know how many [strains] there are now. (Internist 2)

The next subcategory is called “**varying prerequisites for NS1 utilization**” and deals with NS1, a rapid diagnostic test that can capture dengue as early as day 1 of fever and help rule out other diseases. Although the hospitals in Yogyakarta today have access to this tool, utilization of NS1 seems rather arbitrary, according to the informants.

Several clinicians said that the use of NS1 is restricted and applied only in uncertain cases or when it is externally funded and, thus, is free of charge. A few clinicians mentioned cost as an issue but that NS1 could be used if requested by the patient. Another reason for not using NS1 was attributed to the recently established referral regulations and insurance system, which regulate that a patient should first turn to a primary care facility for consultation in the case of fever.

But now there is BPJS and there is a regulation that fever for one to three days should be (treated) at the First Level Health Facilities first. (GP 4)

Other respondents argued that they did not use NS1 owing to the availability of other laboratory tests, although these cannot be used in the early phase of the disease. In fact, it was argued by several informants that there is no point in testing the patient until after day 3:

The earliest I make a diagnosis of whether it is a dengue or not is at day three. Because for me and mostly in real life, we do not make a NS1 test on the first day because for us, clinically, fever has many differential diagnoses—either it is a common cold, sore throat, or something. There are, indeed, many differential diagnoses for that. Therefore, the majority of the doctors will make a dengue diagnosis on day three. (GP 1)

Day four, the lab test will make a differential diagnosis as to whether it is typhoid, whether it leads to dengue, whether it leads to measles. (Pediatrician 1)

Nonetheless, some clinicians seemed to appreciate the potential of NS1 to catch the disease early and showed regret that it was not accessible for everyone, mostly owing to the cost of the test and that it is not always covered by the new insurance system (further explored in the next category).

Actually, for difficulties in diagnosing dengue, it’s just … I think there is no difficulty in diagnosing dengue … You only need to do the NS1 and blood check. Match them to the physical examination or the signs. (GP 2)

Like it or not, we must obey their regulation. NS1 is usually from Puskesmas. (GP 4)

If every single patient with suspected dengue was tested using NS1, it would improve the awareness, because nowadays not every dengue patient comes with classical dengue symptoms. But the problem is the high cost of NS1. Now, we use NS1 for the patient because there is support from the “Elimination dengue program,” which provides free NS1. Otherwise, outpatients have to pay IDR 300,000 which is expensive … (Pediatrician 1)

The last subcategory in this category is called “**inconsistent community attentiveness**” and deals with the importance of a well-informed community and a proper health-seeking behavior to capture the true burden of the disease. The informants seem to be unified in the belief that the local community plays an important role in dengue prevention, but that their current state of knowledge and awareness differed significantly.

One informant expressed the opinion that the situation in the community has become worse in recent years. The reason given was the introduction of the new insurance system, as this was argued to make people more careless and ignorant.

If they are sick, there is Jamkesda [regional health insurance]. So it seems that to visit a hospital in our day, we were afraid of the cost. So parents’ awareness was high, uh, the standard … even health maintenance at home by mothers in the past was good. Children, for example, were not allowed to eat bad food, but now it is ... (Pediatrician 4)

It was also said that doctors frequently have patients with fever showing up with incomplete information and poor awareness of when the fever started, which is important to establish how far the disease has progressed and what tests to use:

... if I met children who had a fever on the first day, but the parents said that they don't have a thermometer, I recommended them to go to the drugstore to buy a thermometer. Fever cannot be judged only through a feeling and by touching with the hand … (GP 1)

These views were contrasted by another informant, who claimed the opposite, that the community is more informed about their diagnostics options today than previously, at least in the urban areas.

Yes, and there are many people who already know now that a laboratory check can be performed earlier. It is an early awareness. That is the trend for the city community because we are talking about the Yogyakarta city area. Urban people here are more like that. But I have no idea about the community members living in the outskirts. (Pediatrician 3)

Nonetheless, most of the clinicians shared the opinion that it is important to keep educating the community about dengue, also with respect to changes in disease trends and patterns relating to changes in weather conditions.

Actually, perhaps more education for the community about how to prevent dengue, what to do after being infected. (Internist 2)

So community perceptions are identical to, “It does not rain anymore. So we don’t need be aware anymore” But this does not guarantee … because the rainy season lasts longer now. (Pediatrician 3)

### Mismatch in regulatory frameworks and interplay with regulatory bodies.

This category is composed of the subcategories “***immature and unequal insurance system***,” “***arbitrary documentation traditions and inappropriate work-arounds***,” and “***friction between regulator and operator****.*” These categories all speak about a health system where certain regulations are not properly synchronized, well known by all stakeholders, or implemented on all levels needed. This includes perceived inefficiencies in the collaboration between the regulatory body (regulator) and executioner of medical care (operator), which is not always seen as optimal. Some of these issues might impose problems on disease diagnostics and influence the local working environment; hence, they have an obvious interaction with the other two categories, as described in Figure 2.

The first subcategory “***immature and inequal insurance system***” directly relates to the introduction of the insurance system called BPJS and challenges imposed by this new regulatory framework.

First, we connect back to the rapid diagnostics test NS1, described in the previous category, as the access and willingness to use this test is significantly connected to the new insurance system, according to the informants. The informants did not give a consistent picture of how the insurance system is implemented in terms of NS1 usage. However, a frequent comment was that NS1 is covered by the insurance only in the case of a positive test and that the test is rather expensive.

In BPJS, the NS1 antigen is covered but the result must be positive. If it is negative, it is not covered. I do not know whether this comes from the BPJS or the hospital. It’s just that we are informed that if NS1 is negative, it cannot be (covered). So in the case of myself and fortunately all the patients whom we have examined, the NS1 was positive so the BPJS could be claimed. (GP 3)

This fact seemed to influence decisions in different ways and indirectly makes access to proper diagnostics unequal. There were clinicians who said that they only offer the test to noninsurance outpatients owing to the high cost, whereas others said quite the opposite: that NS1 is only offered to insurance patients as they consider the cost too high for outpatients not covered by BPJS:

NS1 is expensive. One, it is expensive, uh, and we need see the condition first. Now we have the support of the “Eliminate dengue” program. In the past, there was no support. We do not have the heart to make the patient pay the high cost, it is 300,000 (rupiah) for one patient. And for outpatients, such money is a lot. BPJS obviously does not cover that. (Pediatrician 1)

A few clinicians were not concerned at all with the new insurance system, although their arguments varied. One simply said that the services have not changed as a result of the new regulations, and others reported that it is only when there are changes in the regulatory framework that they encounter problems and that this problem is uninformed patients. Others argued that they will treat the patient regardless, and that the cost and reimbursement schemes are not their concern.

No differences. In fact, it is better because it is clear with BPJS. The regulation is already clear. Patients also know what to bring, the requirements … so it is better. It’s just the challenge when BPJS has new regulations, but it turns out that patients are not informed, not socialized. We are overwhelmed. So ... (MRO 4)

But so far, we have never discharged patients just because the, uh, the BPJS quota runs out. (GP 3)

There were also informants who said that insurance patients with suspected dengue were automatically sent to a higher level hospital and were no longer of their concern. Finally, one informant claimed that the insurance regulations differed between hospitals.

The difference, it’s just, the problem is different hospitals have different rules, ... the BPJS is different at each place ... so it’s not the same. (Internist 3)

The next subcategory “**arbitrary documentation traditions and inappropriate work-arounds**” represents challenges with documentation routines, diagnostic codes, case classifications, and reporting routines, tasks that are of fundamental importance for effective decision-making in disease control.

In line with national regulations, the DHO has established a standard reporting form (called KDRS) that is to be sent in for all laboratory-confirmed dengue cases within 24 hours. In addition, the DHO requires information about all cases, suspected and confirmed. The awareness of the KDRS form, however, varied significantly among the informants, and some clinicians did not even know what it is. There was also a slight confusion about who was responsible for the KDRS report and what was required for this report.

What is KDRS? (GP 2)

There is no report directly made by a doctor, so it is through the nurses. (MRO 2)

... I don’t know who should report that, perhaps the hospital. I just sign it, that’s all. (Pediatrician 4)

Even informants aware of the KDRS routines reported challenges, particularly with respect to poor attitudes or ignorance among the staff. For example, it was reported that certain staff questioned the need for a positive laboratory result (e.g., NS1) to submit the report.

Sometimes specialists do not take our orders (laughing), so they say “No need for this (NS1). It is positive.” But we must make a report. So usually, in staff meetings, there is me and the doctors (laughing). I told them. Let’s just follow (the regulation) because NS1 is also free of charge except for outside of the city government area. Now, praise to God, everyone does what I have said. (MRO 4)

It was also found that people had different perceptions about how strict the 24-hour rule is and that reports were rarely sent within 24 hours to the DHO. Several clinicians said that the KDRS is written and submitted when the patient is already at home, or ready to return home. It was argued that this would allow the laboratory results to be up-to-date and that the distinction between DF and DHF could be made. This indicates that the report might be submitted several days after the initial diagnosis was made. The main argument for late reports were, however, that instead of submitting the KDRS report they would take a picture of the report, including patient information and full address, and share instantly with the DHO and other health facilities using the social media platform WhatsApp. The KDRS report could thus follow a few days later without compromising the 24-hour regulation.

The DOH gives us, what do you call it, uh, a time period, if it is possible, do not report it more than a week later, but it is better to deliver it within 1 × 24 hours. (MRO 2)

It does not have to be within 1 × 24 hours but if a DHF diagnosis is already made, we send it by WhatsApp. (MRO 1)

Yes. But at least if we want it to be quick, my colleagues will take a picture of it and send it immediately. (MRO 4)

Finally, there was no consensus found regarding what diagnostic codes or case classification to use for diagnostics and reporting among the informants. According to the DHO, ICD10 codes and the WHO 2009 guidelines were implemented, and, thus, the new case classifications shall be used (dengue with or without warning signs and severe dengue). Nonetheless, the KDRS report applies the old case classifications, DF, DHF, and DSS. Some clinicians knew that all dengue cases should be reported, whereas others claimed that only the severe cases or deaths were to be included in the report. There were also informants who explained that changes have been made recently to include DF as well, but that these cases were not reported previously.

Talking about the coding, we only follow the diagnosis written on the medical report document. So between DHF and DF, the coding will be different. There is no difficulty. (MRO 2)

So, uh, sometime there is a miscommunication with NS1. NS1 from the DOH is already, uh, what is it, decided, I mean if the NS1 is positive, IGG, IGM is positive, it is a dengue. It’s just there is a doctor who writes the NS1 positive but it is included into DF. Like it or not, we follow the doctor. It’s just later when we report in the WhatsApp, that we will inform DF with NS1 positive. (MRO 3)

It is important because with any dengue, DHF, I report them all, even if it is just a DF. (Pediatrician 3)

Questions about the use of codes in general gave very vague answers, but most of the respondents answered that they used the old case classifications. Questions addressing ICD codes specifically were met mainly with defensive replies. There were, however, occasions where the new case classifications were implemented in the clinical pathways.

Frankly speaking, Miss […] I do not follow ICD that much. (Internist 3)

I use the WHO (2007) one more ... [ ] Yes, but recently after reeducation, we use WHO 2011. (GP 1)

Government policy influences it too. So sometimes, perhaps based on 2011, it is DF or something else. The report changes too. (GP 4)

Despite the variable compliance with recommended routines reported so far, there were hospitals where the system of diagnosing and reporting dengue cases to the health authority was considered to be functioning well. Moreover, most informants communicated a very positive attitude toward the reporting requirements, arguing that this information is very important for policymaking and dengue prevention in general.

For the eight months I have worked here, praise to God, there is no delay. The coordination is still good. (MRO 4)

Based on my science, it is important because once more I have to say that policy is based on data. Therefore, even though people still see it as unimportant, in my personal opinion, scientifically, it is very important that we know the number of incidents, the location mapping, then the age distribution, and others. (GP 1)

The last subcategory is called “***friction between regulator and operator***” and represents collaboration and communication challenges between the local health authority and the hospital staff, and the perception that the health authority needs improvement in certain areas to make the clinical work more efficient.

A few clinicians reported a feeling that the DHO does not respect or understand the nature of clinical work. For example, it was said that the DHO sometimes scheduled meetings at a very short notice. This makes it difficult for the staff to make time for the meeting, which was at the same time seen as an important opportunity for knowledge updates.

What I dislike about them is that they schedule a meeting at the last minute, for an example, there is a meeting tomorrow, and I have only been informed today. For them, perhaps three days is not seen as the last minute for sending an invitation. But for us in the field, we’re called at night “Doc, there will be a meeting tomorrow …” (Pediatrician 1)

Another criticism raised was the lack of outbreak information from the health office, which was considered important information for the medical staff to make proper clinical judgments. Although not raised by more than a few without being asked, some clinicians said that they do not receive outbreak information from the DHO when asked directly. Others did report that they receive outbreak information, but that these were sent using WhatsApp like the KDRS case reports.

Moreover, I just read on Tribun that in Jogja there are already 11 who died because of dengue. Eleven died because of dengue but we never heard about that. (GP 3)

Outbreak warnings are given but they are sent via WhatsApp groups. (MRO 2)

No. The information is from myself. From my patients, there is no information that it’s increasing. (Internist 2)

It was also mentioned that the local health office fails to follow-up other important diseases with similar initial symptoms to DF, such as leptospirosis. This is one of the DDs, and information about such cases might be of importance for proper clinical judgment.

The City (DOH) needs an improvement. Other areas have leptospirosis but they never … (They) never ask for any report (about it). (Internist 3)

Several informants did consider the relationship with the local health government to be good, although with the potential to improve. Others expressed that they have indeed received feedback on their work. All administrative staff mentioned that there were regular follow-ups at the health office or the hospital—for example, the local disease surveillance team and regular discussions on WhatsApp to reach consensus about the current disease situation.

My advice is that this should stay good as it is now. Now it is already good. Hopefully it will be better and can be the best. (MRO 4)

Before we meet, usually we discuss the issues on WhatsApp. (MRO 1)

Yes, we get information about the outbreak from DHO, also we were informed that our hospital got an appreciation from DHO regarding the best hospital on reporting. (MRO 4)

### Different prerequisites at different hospitals.

This last category is composed of the subcategories “**standard operating procedure not standardized**” and “**inconsistent conditions for knowledge updates.**” This category represents the finding that there are sometimes large variations between the hospitals’ internal capacity in managing dengue and keeping staff informed and updated.

The first subcategory “**standard operating procedure not standardized**” represents the situation where hospitals follow different processes internally and have different so-called standard operating procedures, standard operating procedures (SOPs), for managing DF in their clinic.

According to most clinicians, their hospital has a standard operating procedure for dengue, sometimes called a “clinical pathway,” but there were those that replied that they have no such routine in place. In cases where an SOP was indeed implemented, it was typically developed in-house and not provided by a regional or national health authority, although there were indications that there might be such guidelines available.

Yes. I developed it some time ago, based on a guideline when I was studying. (GPs 3)

I haven’t had the most recent one from the Ministry of Health. But I have developed my own SOP. This is an SOP for treating suspects of dengue fever and DHF in hospital. (GP 3)

The information sources used to develop the local guidelines appeared to vary as well. Most of the clinicians referred to the WHO guidelines, and a few referred to external seminars provided by a specific research project and a national pediatrics association:

There is no SOP from [hospital name] but my guideline is from WHO. Book, uh, WHO children service standard… (GP 3)

Dengue treatment for children is based on the one from WHO and SPM (Standar Pelayanan Minimal/Minimum Service Standard) and from IDAI (Ikatan Dokter Anak Indonesia/Indonesia Pediatrician Association). (Pediatrician 2)

[I] got this from a seminar. There was a seminar given by the public health research department … [ ] ... They gave us such information and they also said that it would be better if doctors at … general practitioners are provided with an SOP. (GP 3)

Although the SOPs vary between the hospitals, compliance with the SOPs were said to be monitored regularly, according to most respondents, either by a “clinical pathway team” or senior staff.

We do as the SOP says. It should be resuscitating the liquid then watching for the DSS signs, is there any ascites, etc … (Pediatrician 1)

So, the decision making is still the doctor in charge of the patient. Even though the clinical pathway says this, but if the doctor in charge wants to say, “No, this can’t be done. The examination, for example, just do the NS1. Oh, this one is only thrombosis,” we will just obey the specialist. So, it cannot be forced. This is difficult, sometimes. (GP 4)

Finally, the existence of multiple non-standardized guidelines was directly attributed as being a higher risk for the patients. Some clinicians said that a lack of standardization of laboratory cutoffs, for example, made less-educated colleagues more likely to make errors, and matters were made worse by the fact that doctors sometimes work in more than one hospital.

So far, they still think that the platelet is the most important. So, if the platelet is still high even though the hematocrit is already increased, they still make them an outpatient...Probably that is the mistake. The unstandardized value about the platelet. (Internist 2)

Also, about different regulations because they practice in three places. Something like that. If it happens here, we warn them. They said, “Usually in hospital A, the patient is not hospitalized, Doc.” It turns out that they also work in other hospitals and they have different regulations there. (Pediatrician 2)

The last subcategory, “**inconsistent conditions for knowledge updates**,” describes unequal opportunities and varying willingness to keep updated on dengue epidemiology, diagnostics, treatment, interventions, or regulatory matters.

Staying updated was indeed considered very important for most of the clinicians, although their arguments varied. Most clinicians argued that they have to attend seminars or classes as part of their professional development, whereas others simply said that they must stay in touch with the latest development in the field as the region they work in is endemic and dengue cases remain high despite all efforts. Several clinicians also talked about the fact that knowledge fades and must be updated regularly.

It will be very helpful, to be reminded again, to refresh again. It’s all that’s needed. Refresh. Yes, refreshing because deterioration is there. We have spent a long time outside of the university, so it is there for sure. (GP 4)

If I do not update my knowledge, I will be left far behind. When there is a patient, the patient cannot be cleverer than the doctor, right? (GP 3)

It is necessary because we live in an endemic area. There must be new findings, must be new therapies, new treatment methods. We need to know them. (GP 3)

It is one of the SIP (Surat Ijin Praktik/Practice License) requirements in order to have a practice. So, like it or not, we must update. (Internist 2)

The opportunities for staying updated varied with workplace as well as profession, and the preferred source for new knowledge also appeared to differ. Some clinicians said that they use the Internet, read journals, or attend external seminars. However, seminars were said to come with a cost, and several clinicians suggested that they should enable knowledge upgrades with the help of the internal specialists instead. It was also suggested by several informants that the training should include all staff, not only doctors. District health office seminars were mentioned frequently as a source of new knowledge, although criticism was raised that these meetings are not available to everyone and sometimes scheduled at too short notice, as mentioned in the previous category.

Attending seminars, sometimes the DOH itself invite hospitals, doctors. Doctors usually perfer symposiums. Pediatricians, internists are included in tropical diseases, so usually it is presented by tropical medicine. (GP 4)

Usually, the training is from the training and education department using a doctor as a resource person. For us general practitioners, we do have a doctor group and we often share knowledge there. (GP 1)

For me, because now it is all about digital gadgets, so we use the Internet for browsing about dengue. Also search for journals and information. (Pediatrician 3)

I mean my suggestion is that this hospital should facilitate its doctors to update their knowledge. There is no need to send us to seminars outside, we can use our internal doctors—for example, the internists and the pediatricians … Not just the doctors, it can also be the nurses, it can be everyone. (GP 3)

Several of the clinicians indicated that the work of keeping their knowledge updated was up to each doctor, and a few of them mentioned that they could request an update in their workplace if they wanted to. Attitudes were thus to blame for knowledge discrepancies according to some clinicians, who claimed that some doctors are less interested in updating their knowledge than others.

But doctors mostly update their own knowledge by themselves. (GP 3)

For knowledge, it seems for doctors it is almost similar. It just depends whether he/she wants to update or not. (GP 4)

Perhaps most doctors, maybe, there are some who tend, uh, I know more about the senior doctor. That’s number 1 [name], number 2 [name], they may be too lazy to update their knowledge. (Pediatrician 1)

It was also found that the opportunities to pass on knowledge, newfound or not, to colleagues varied greatly among the hospitals. Some mentioned regular weekly or daily meetings to update colleagues on special cases, diseases, or the current status in the clinic. However, several clinicians said that there was no such opportunity in their workplace but that it was desired. Others mentioned that there were regular meetings at their workplace, but the opportunity for people to attend these meetings seemed to vary.

So far, no we don’t [share internally]. (GP 3)

Presenting, yes. Moreover, if we are sent to the DHO. (GP 4)

Yes, we have that. Every Thursday, all general practitioners, all medical staff, and specialists are gathered and do a morning report. It is the general practitioners who do the report. If there are cases like the one I mentioned, we tell them what to do. (GP 2)

As far as I know, they have a morning report, but I am unavailable in the morning. It’s the same as in [hospital name]. I’m unavailable in the morning too. (Internist 3)

Moreover, few clinicians claimed that reading was not enough and that more should be done to increase the standard of the medical profession in terms of dengue management.

Clearly, we have new knowledge and old knowledge … Perhaps, this is my personal opinion, if we want to treat a DHF case, we must have a certificate or training because if we just get it from reading, it seems that it is not tested. (Pediatrician 1)

## DISCUSSION

It is a well-known fact that diagnosing febrile and infectious diseases can be difficult and particularly challenging in countries that are affected by multiple diseases.^26^ Indonesia is no exception. The health system in Yogyakarta frequently deals with a multitude of infectious diseases, including chikungunya, leptospirosis, typhus, and common flus, many of which are DDs for dengue. Consequently, the inherent difficulties in diagnosing the disease were found to be a notable challenge for the disease surveillance system under study, particularly as the pattern of disease was claimed to have changed in recent years. The term “expanded dengue” was thus frequently used by the informants. A change in disease pattern has indeed been documented elsewhere,^27,28^ and it has been found that dengue comes with a wider spectrum of symptoms than previously observed.^29–31^

To overcome the diagnostic challenges mentioned, a solid system must be in place, which gives the clinicians the best possible prerequisites for making the correct diagnosis and, thus, the health authorities a correct picture of the disease burden. This includes a standardized regulatory framework for diagnostics and case reporting, access to the best diagnostic tools, staff with proper qualifications, and a well-functioning leadership and governance. In addition, to capture the true burden of the disease, a well-informed local community with a proper health-seeking behavior is essential.^32^

From a helicopter view, most of the components of a well-performing dengue surveillance system were indeed already available, although not in all hospitals. In line with a recent review of the decentralized Indonesian health system,^33^ our study found issues in several of the building blocks of a well-performing health system as defined by the WHO.^34^

First, it was found that the regulatory frameworks used to support the staff in diagnostics and reporting were inconsistent. Ideally, this framework consists of the following components: 1) a clinical pathway or SOP (stipulating what should be done with the patient and when), 2) documentation routines (stipulating what case classification and, thus, diagnostic codes to use), and 3) case reporting routines (stipulating when and how dengue cases are reported to the health authority).^35–37^ According to the analysis, there seems to be no or minimal coordination between these routines, and, in some places, there appeared to be no standardized routines at all established. The only exception was the case reporting routine (KDRS), which was available in all hospitals. The KDRS is further the only routine regulated and enforced by the DHO. In places where there was an SOP available, it was frequently found that it was based on the WHO guidelines (2009 or 2011) as recommended by the National Ministry of Health, although developed in-house. However, this imposes another problem, as the KDRS asks for the old case classifications DF, DHF, and DSS, whereas the ICD10 codes and the WHO guidelines from 2009 and 2011 classify cases according to probable dengue—with or without warning signs—and severe dengue. These guidelines have been highly debated previously, and there are numerous studies that have argued the pros and cons of the various case definitions.^38–40^ Surprisingly, none of the informants brought this to our attention, but this discrepancy might very well explain some of the confusion that was found regarding reporting requirements and whose responsibility it was to file the reports. This potential issue is likely to be further aggravated by the fact that doctors can work in more than one hospital according to Indonesian law.^41^

Another intriguing finding was that the social media platform WhatsApp was used to communicate dengue-related information to various stakeholders to improve transparency and lead times from diagnostics to reporting. This social media platform is widely used across Indonesia but has been restricted recently and was even shut down for some time during spring 2019 (dengue season), according to global news media.^42^ Especially worrisome was the finding that this platform was used to submit KDRS reports, which contains patient information, including name and address. Although this “work-around” was seen to significantly improve the likelihood that the dengue reports were sent to the health authority within 24 hours, building routines on this kind of platform cannot be recommended. The personal data protection law in Indonesia is still considered weak, although a new regulation is under development by the Indonesian government.^43^ WhatsApp was also mentioned as being used to provide outbreak information, according to some informants. This is a less sensitive topic, and the information can be of value for other stakeholders as well, including the community when they are included in the information loop.^44^ Social media has, in addition, been shown to be a promising real-time proxy for disease events^45^ and to enhance outbreak predictions compared with the use of routine data alone.^46,47^

Second, a high risk of inequalities in the care provided was identified owing to the aforementioned regulatory issues, as well asthe introduction of the new insurance system, BPJS.^19^ The system was by many considered insufficient, especially with respect to dengue, as the BPJS obviously fails to cover the cost of the NS1 test. It was also found that NS1 was used quite arbitrarily, and two informants expressed a wish for every suspected case to have the NS1 test. Concern has, however, been raised that such screening would become a burden to the health system owing to the resources required, both in terms of staff and money. Already now, the cost for each dengue case is estimated to be 791 USD in Yogyakarta and almost twice this amount in Jakarta and Bali.^48^ Hence, improving the capacity of the staff to make a qualified judgment on who needs the test might be a better solution, which brings us to the third concern raised by this article—the quality of the staff and their access to continuous education on dengue-related matters.

There appear to be differences in both the opportunity for and willingness to participate in educational activities aimed at updating the staff knowledge about dengue and other diseases.^49^ Some of the hospitals appear to have good routines for keeping their staff up to date, for example, through case reviews, whereas others express a wish for improvement in this area. Seminars arranged by the DHO appear to be an appreciated source of new knowledge but apparently are not available to everyone.

This study had some limitations, which should be considered when interpreting the findings. First, the respondents were not randomly chosen, and neither were the hospitals included in the study. Hospitals were chosen by the researcher, and respondents were appointed by the hospital management. Nonetheless, saturation was reached, and the material has been found to be rich in nuances and cover many important aspects and opinions. Another potential weakness is that the material has been processed and analyzed in two different languages; hence, important meanings might have been lost in translation.

## CONCLUSION AND RECOMMENDATIONS

In the light of the global expansion of dengue and observed changes in the disease pattern, and also with respect to the season and duration of epidemics in endemic regions, there is a need to further strengthen the dengue surveillance system, not only in Yogyakarta. After all, it is a well-known fact that dengue is severely underreported globally, although little has been done to explore why.

This article has studied the foundation for the dengue surveillance system in an endemic region of Indonesia, and concrete improvement potential has been found that could be applied to the Indonesian health system, and beyond. It is a strength that most building blocks are already in place and the greatest potential is seen in the streamlining of regulatory frameworks, including synchronizing local and national/international frameworks, such as the KDRS report and the diagnostics guidelines. However, having a good surveillance system itself is not sufficient when it cannot respond and deliver an alarm for the prevention purposes. In addition, the local health authority is recommended to facilitate the implementation of standardized SOPs across all hospitals, and to support the hospital management in their work to enable people to gain access to the most recent updates and routines established.
